# Transgenic Overexpression of 14-3-3 Zeta Protects Hippocampus against Endoplasmic Reticulum Stress and Status Epilepticus *In Vivo*

**DOI:** 10.1371/journal.pone.0054491

**Published:** 2013-01-24

**Authors:** Gary P. Brennan, Eva M. Jimenez-Mateos, Ross C. McKiernan, Tobias Engel, Guri Tzivion, David C. Henshall

**Affiliations:** 1 Department of Physiology and Medical Physics, Royal College of Surgeons in Ireland, Dublin, Ireland; 2 Department of Biochemistry, University of Mississippi Medical Center, Jackson, Mississippi, United States of America; Johns Hopkins University, United States of America

## Abstract

14-3-3 proteins are ubiquitous molecular chaperones that are abundantly expressed in the brain where they regulate cell functions including metabolism, the cell cycle and apoptosis. Brain levels of several 14-3-3 isoforms are altered in diseases of the nervous system, including epilepsy. The 14-3-3 zeta (ζ) isoform has been linked to endoplasmic reticulum (ER) function in neurons, with reduced levels provoking ER stress and increasing vulnerability to excitotoxic injury. Here we report that transgenic overexpression of 14-3-3ζ in mice results in selective changes to the unfolded protein response pathway in the hippocampus, including down-regulation of glucose-regulated proteins 78 and 94, activating transcription factors 4 and 6, and *Xbp1* splicing. No differences were found between wild-type mice and transgenic mice for levels of other 14-3-3 isoforms or various other 14-3-3 binding proteins. 14-3-3ζ overexpressing mice were potently protected against cell death caused by intracerebroventricular injection of the ER stressor tunicamycin. 14-3-3ζ overexpressing mice were also potently protected against neuronal death caused by prolonged seizures. These studies demonstrate that increased 14-3-3ζ levels protect against ER stress and seizure-damage despite down-regulation of the unfolded protein response. Delivery of 14-3-3ζ may protect against pathologic changes resulting from prolonged or repeated seizures or where injuries provoke ER stress.

## Introduction

14-3-3 proteins are a ubiquitous family of molecular chaperones of which seven isoforms are known in mammals (β, ε, γ, η, ζ, θ, and σ). 14-3-3 proteins regulate cell proliferation, differentiation, metabolism and apoptosis [1]. 14-3-3 proteins are present within synapses and are important for the function and localization of ion channels [2]. 14-3-3 proteins can also function as sweepers of misfolded proteins [3] and promote protein trafficking from the endoplasmic reticulum (ER) [4]. Genetic deletion studies have demonstrated non-redundant roles for certain isoforms in brain development and function [5], [6] while aberrant expression of 14-3-3 proteins has been implicated in several diseases of the nervous system [2].

Prolonged seizures (status epilepticus) or repeated seizures over time (pharmacoresistant epilepsy) can damage the brain [7]. Excitotoxicity is a key mechanism, whereby prolonged over-activation of glutamate receptors results in loss of intracellular calcium homeostasis, oxidative stress, damage to intracellular organelles, and necrosis [8], [9]. Seizures also trigger release of apoptogenic proteins from mitochondria and downstream caspase-dependent and -independent neuronal death [10], [11]. 14-3-3 proteins may be important upstream regulators of apoptosis-associated signalling after seizures. 14-3-3 proteins dissociate from pro-apoptotic proteins such as Bad and apoptosis signal-regulating kinase 1 (ASK-1) after experimental status epilepticus, promoting neuronal death [12], [13], [14]. While some 14-3-3 isoforms are down-regulated after seizures [12], [15], levels of the zeta (ζ) isoform are increased [15], [16], [17]. This may be neuroprotective since depleting 14-3-3ζ levels *in vitro* exacerbates kainic acid excitotoxicity [17]. Reduced 14-3-3ζ expression was reported during epilepsy development [18] and 14-3-3ζ is also involved in the function of tuberin, mutations in which result in neurological phenotypes including seizures [19].

Although 14-3-3 proteins are mainly cytosolic they are also found in the ER-containing microsomal fraction [15], [20]. ER functions include regulating protein folding and trafficking and intracellular calcium storage [21]. Cell stress can result in the three-branched unfolded protein response (UPR) [22]. Ire1, an endoribonuclease, cleaves the X-box binding protein 1 (*Xbp1)* transcript resulting in upregulation of molecular chaperones such as Bip (glucose-regulated protein 78/GRP78); cleavage of activating transcription factor 6 (ATF6) leads to increased ATF4 levels and modulators of ER stress; activation of protein kinase RNA (PKR)-like ER kinase (PERK) which phosphorylates eukaryotic initiation factor 2α (eIF2α) leading to a shut-down in protein translation. If ER stress persists, the ATF6 and PERK branches trigger apoptosis through up-regulation of CHOP and activation of caspases [23]. ER stress may be an important pathophysiological component in experimental and human temporal lobe epilepsy (TLE) [24], [25], [26], [27], [28]. Moreover, inhibition of ER stress can protect against seizure-induced neuronal death [25], [29].

Whether 14-3-3ζ can protect the hippocampus against either ER stress or seizure-induced neuronal death *in vivo* is unknown. To test this idea we studied ER stress and the response to seizures in transgenic mice over-expressing 14-3-3ζ.

## Methods

### 14-3-3ζ Mice

Generation of 14-3-3ζ-overexpressing mice (hereafter referred to as 14-3-3ζtg) has previously been reported [30]. The SJL mice express myc-tagged mouse 14-3-3ζ under the control of the ubiquitous elongation factor 1α (EF1α) promoter. Transgene expression was confirmed by Western blot analysis of the myc tag in protein lysates from tail snips, as described [30]. Heterozygous males and wild-type females were bred together to obtain heterozygous 14-3-3ζtg and wild-type littermate controls. Mouse body and brain weight was recorded in 6 week old animals.

### Seizure Model

All mouse experiments were performed in accordance with the European Communities Council Directive (86/609/EEC) and were reviewed and approved by the Research Ethics Committee of The Royal College of Surgeons in Ireland (REC#205) under license from the Department of Health, Dublin, Ireland. Food and water was available to mice *ad libitum*. Induction of status epilepticus in SJL wild-type and 14-3-3ζtg mice was performed as previously described [31]. Briefly, mice were anaesthetised with isoflurane and placed in a mouse adapted stereotaxic frame. After making a mid-line scalp incision, three partial craniectomies were performed for placement of skull electrodes. A fourth full craniectomy was drilled for the placement of a guide cannula (Coordinates from Bregma: anterior-posterior (AP) = −0.3 mm and laterally (L) = −0.3 mm) [32]. The cannula and electrodes were fixed in place and mice allowed to recover before being placed in a Perspex container. The EEG was recorded using a Grass Comet Digital EEG. After baseline EEG was established, an injection cannula was lowered through the guide cannula 3.75 mm below the brain surface for the injection of kainic acid (Sigma-Aldrich) into the basolateral amygdaloid nucleus. After 40 min all mice received an i.p. injection of lorazepam (6 mg/kg) to curtail seizures and reduce mortality and morbidity. For i.c.v injections, mice were fitted with a cannula as described [33] and received 1 µl injection of 50 µM tunicamycin (Sigma-Aldrich).

EEG was analysed using Grass software with additional frequency and amplitude analyses of EEG data performed by uploading the data to an automated EEG analysis programme (LabChart pro v7 software, ADInstruments Ltd) [33]. Seizures were defined as the duration of high amplitude (>2×baseline) high frequency (>5 Hz) discharges and calculated between the time of KA injection and lorazepam [33].

### Hippocampal Primary Neuron Culture

Hippocampal primary neurons were cultured as previously described [34]. Briefly, neurons from wt or 14-3-3ζtg E18 embryos were cultured for 6 days and then exposed to 3 µM KA for 24 h followed by assessment of cell death by propidium iodide staining. Cell death was expressed as a % of total cells.

### Western Blotting

Hippocampus was microdissected to obtain CA1, CA3 and dentate gyrus enriched portions, as described [33], [34]. For analysis of basal levels in naïve mice, subfields were pooled from both hemispheres. Samples were homogenised in lysis buffer containing 0.01 M Tris-HCl and 1 mM EDTA. Proteins were separated by SDS-polyacrylamide gel electrophoresis, transferred to nitrocellulose membranes and then incubated with antibodies as follows; Tubulin (Sc-8035, 1∶10,000), 14-3-3 ζ, β, ε, η, γ, θ (Sc-1019, -1657, -135816, -17286, -731, -59414, 1∶5,000), GluR6/7 (Sc-28797, 1∶2,000), Calnexin (Sc-11397, 1∶1,000) (All Santa Cruz Biotechnology), Bax (2772, 1∶500), Bim (2819, 1∶100), Lamin A/C (2032, 1∶500), p-eIF2α (9721, 1∶1,000), Caspase-12 (2202, 1∶1,000) (all Cell Signaling Technology), NeuN (MAB377, 1∶400), GFAP (AB1540, 1∶1,000), KA2 (06-315, 1∶2,000), KDEL (420400, 1∶500) (all Millipore), Bad (ab32445, 1∶500), Iba1 (ab5076, 1∶1,000), Porin (ab15895, 1∶4,000), ATF4 (ab23760, 1∶500) (all Abcam), ATG5 (NB110-53818, 1∶1,000) (Novus Biologicals). Membranes were next incubated with HRP –conjugated secondary antibodies (1∶2,000 Millipore) and bands visualised using Imobilon western HRP substrate (Millipore) using a FujiFilm LAS-4000 system under chemiluminescence.

### RNA Isolation, cDNA Synthesis and Real-time PCR

Total RNA was extracted from brain samples using Trizol as previously described [35]. First strand cDNA synthesis was performed using 1 µg of total RNA and SuperScript II reverse transcriptase (Invitrogen) primed with 50 pmol of random hexamers. Quantitative real-time PCR was performed using the LightCycler (Roche Diagnostics, Basal, Switzerland) and the QuantiTech SYBR Green PCR kit (Qiagen) according to manufacturer’s protocol. Specific primers for each gene assayed were purchased from Sigma and sequences were as follows; 14-3-3ζ; (Fw5′-ACCGTTACTTGGCCGAGGTT-3, Rv5′-GCAGGCTTTCTCTGGGGAGT-3′), 14-3-3γ; (Fw5′- CAGCAGCATCGAGCAGAAGA-3′, Rv5′-CTGGGTCTCGCTGCAGTTCT-3′), 14-3-3ε (Fw5′- GGGAGTACCGGCAAATGGTT-3′, Rv5′-TCTGCTGCCTCCTTCCTGTC-3′), Grp78 (Fw5′ TGCAGCAGGACATCAAGTTC-3′, Rv5′-CTGCATGGGTGACCTTCTTT-3′), Xbp1s (Fw5′- GAGTCCGCAGCAGGTG-3′, Rv5′-AAGGGAGGCTGGTAAGGAA-3′), β-actin (Fw5′- AGGTGTGATGGTGGGAATGG-3′, Rv5′-GGTTGGCCTTAGGGTTCAGG-3′). The data were normalised to β-actin and relative mRNA transcript levels were quantified using the standard ^ΔΔ^CT method.

### Subcellular Fractionation

Hippocampi were fractionated to obtain the cytoplasm, mitochondria, nuclear and microsome-enriched fractions, according to previous methods [15]. Briefly, samples were homogenized in a mannitol/sucrose buffer containing a protease inhibitor cocktail and then centrifuged twice at 1200×*g* for 10 min. The post-nuclear supernatant was then centrifuged twice at 10 000×*g* for 15 min and the resulting mitochondrial pellet was resuspended in a sucrose buffer and purified through a percoll bilayer by centrifugation at 41 000×*g* for 30 min. The crude cytosolic fraction was then centrifuged at 100 000×*g* for 1 h to separate the microsomal and cytosolic fractions. Following fractionation, protein samples (20 µg) were boiled in gel-loading buffer and then separated by 12% SDS-PAGE. Proteins were transferred to nitrocellulose membranes and then incubated with appropriate antibodies. Membranes were then incubated with appropriate secondary antibodies (1∶2000 dilution) followed by chemiluminescence detection on a FujiFilm Las4000 imager.

### Histopathology and Immunohistochemistry

Fresh-frozen brains were processed on a cryostat in the coronal plane and 12 µm sections collected at the level of dorsal and ventral hippocampus [31]. Sections were nissl-stained using standard techniques [36]. DNA fragmentation was analyzed using terminal deoxynucleotidyl dUTP nick end labeling (TUNEL) (DeadEnd Fluorometric TUNEL system, Promega) as described [34]. Irreversible neuronal injury was assessed using Fluoro-Jade B (FJB) staining [34]. Briefly, sections were post-fixed, incubated in 0.006% potassium permanganate, rinsed and transferred to 0.001% FJB solution (Chemicon Europe Ltd., Chandlers Ford, UK). Sections were then rinsed, dried, cleared and mounted in DPX (Sigma–Aldrich). For immunohistochemistry, sections were post-fixed, permeabilized, blocked in 5% goat serum and incubated overnight with antibodies against; NeuN (1∶400) and KDEL (1∶500) (Millipore). Sections were washed and incubated with secondary antibodies conjugated with AlexaFluor 488 for NeuN or AlexaFluor 568 for KDEL (Biosciences Ltd). Sections were mounted with medium containing DAPI (Vector Laboratories Ltd) and examined using a Hamamatsu Orca 285 camera attached to a Nikon 2000s epifluorescent microscope. Counts were performed under 20× magnification lens with a counting window area of 725 µm×550 µm and were the average of those from two adjacent sections.

For 14-3-3 immunohistochemistry, free floating coronal sections were stored in a 24 well plate in an anti-freeze solution containing ethylene glycol. Sections were treated with 1% H_2_O_2_ to deactivate peroxidises and then blocked with 10% BSA/FBS solution for 90 min. The following antibodies were then applied to sections overnight at 4°C; 14-3-3 ζ, γ (1∶1,000, Santa Cruz Biotechnology), ZnT3 (1∶100, Synaptic Systems). Sections were then incubated with biotinylated antibodies with horse/donkey serum for 90 min before being treated with Avidin ABC peroxidise complex for 1 h. Sections were washed then incubated with DAB, washed again, mounted and coverslipped. For immunofluorescence microscopy, free-floating sections were stained with anti-myc and then either NeuN or GFAP followed by either AlexaFluor 488 or AlexaFluor 568–conjugated secondary antibodies.

### Data Analysis

All data are presented as mean ± standard error of the mean. Gel densitometry was undertaken using gel-scanning integrated optical density software (AlphaEaseFC v4.0). Two group comparisons were made using Student’s *t* test (GraphPad Instat). Significance was accepted at *p*<0.05.

## Results

### Distribution of myc-14-3-3ζ Transgene in the Mouse Brain

**Figure 1:**
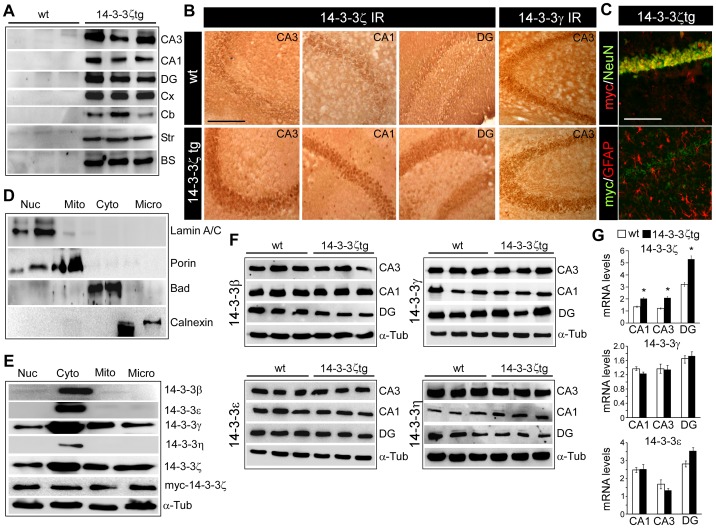
Distribution of the myc-tagged 14-3-3ζ transgene in mouse brain. (A) Western blot analysis (*n* = 1 per lane) of microdissected brain regions from wild-type (wt) and 14-3-3ζtg mice immunoblotted with antibodies against the myc tag confirm expression of the transgene in CA subfields, dentate gyrus (DG), cortex (Cx), cerebellum (Cb), striatum (Str) and brain stem (BS). (B) 14-3-3ζ immunostaining in hippocampal tissue sections showing higher immunoreactivity (IR) in 14-3-3ζtg compared to wt mice. Panels to the far right show sections stained for 14-3-3γ revealing normal neuronal distribution and level between wt and 14-3-3ζtg mice. (C) Representative fluorescence immunostaining of the CA1 subfield showing (top) myc (red) with NeuN (green) co-localization and (bottom) myc (green) with GFAP (red) confirming mainly neuronal expression of the transgene. (D) Immunoblots of SJL mouse hippocampal fractions (*n* = 1 per lane) for the select presence of markers of the nucleus (Nuc), mitochondria (Mito), cytoplasm (Cyto) and microsome-containing ER fraction (Micro). (E) Western blots from pools of 14-3-3ζtg mouse hippocampi (*n* = 1 per lane) show the presence of various isoforms in different compartments. Note, endogenous 14-3-3ζ and myc-14-3-3ζ are similarly distributed in each fraction. (F) Protein levels of various 14-3-3 isoforms in wt and 14-3-3ζtg mice hippocampal subfields (*n* = 1 per lane). (G) Real-time PCR measurement of ζ, γ and ε 14-3-3 isoform levels in wt and 14-3-3ζtg mice (*n* = 3 per group). *p<0.05 compared to wt. Scale bars in B, C, 160 µm.

Heterozygous transgenic mice overexpressing myc-tagged mouse 14-3-3ζ under the EF-1α promoter on a SJL background were bred and genotyped as before [30]. Mice were born at expected rates and developed normally. EF-1α is constitutively expressed in the brain and we began by characterizing the distribution of the 14-3-3ζ transgene in different brain regions, by western blot detection of the myc tag (Figure 1A). Myc-14-3-3ζ was present in all major subfields of the mouse hippocampus, at its expected molecular weight of 34 kD (representing the 28 kD 14-3-3ζ protein plus the 6 kD myc tag). Myc-14-3-3ζ was also detectable in the neocortex, cerebellum, striatum and brain stem (Figure 1A). To support these data we stained tissue sections from wild-type and 14-3-3ζtg mice using antibodies against 14-3-3ζ (Figure 1B). Endogenous 14-3-3ζ was detected mainly in neurons in the CA pyramidal layers and in granule neurons in the dentate gyrus, in a somal distribution (Figure 1B). Higher immunoreactivity for 14-3-3ζ was evident in all hippocampal subfields in 14-3-3ζtg mice (Figure 1B). We did not detect significant 14-3-3ζ immunoreactivity in glia. Immunoreactivity for 14-3-3γ was not different between genotypes (Figure 1B, far right panels). Double immunofluorescence staining of hippocampal sections for myc and either NeuN or glial fibrillary acidic protein (GFAP) confirmed the transgene was primarily expressed in neurons (Figure 1C).

We next examined subcellular fractions from the hippocampus of wild-type and 14-3-3ζtg mice to determine if the myc-14-3-3ζ distributed normally within cells. Immunoblotting using specific markers confirmed purified nuclear, mitochondrial, cytosolic and ER-containing microsome fractions (Figure 1D). The β, ε and η isoforms were present exclusively in the cytosolic fraction of the mouse hippocampus (Figure 1E). In contrast, 14-3-3ζ and 14-3-3γ were found in all cellular compartments (Figure 1E). The myc-tagged 14-3-3ζ was also present in each subcellular fraction (Figure 1E).

### 14-3-3ζ Over-expression does not Produce Compensatory Changes in Other 14-3-3 Isoforms

We next investigated whether over-expression of 14-3-3ζ led to compensatory changes to levels of other 14-3-3 isoforms. Western blot analysis of microdissected hippocampus revealed normal levels of various 14-3-3 isoforms, including 14-3-3β, 14-3-3ε, 14-3-3γ and 14-3-3η, in 14-3-3ζtg mice (Figure 1F), indicating that overexpression of 14-3-3ζ does not produce changes to other 14-3-3 isoforms in the hippocampus. Real-time PCR analysis confirmed ∼two-fold higher 14-3-3ζ transcript levels in transgenic mice in all hippocampal subfields whereas there were no mRNA differences in expression of other 14-3-3 isoforms in the hippocampus of 14-3-3ζtg mice (Figure 1G and data not shown).

### Normal Hippocampal Morphology in 14-3-3ζtg Mice

**Figure 2:**
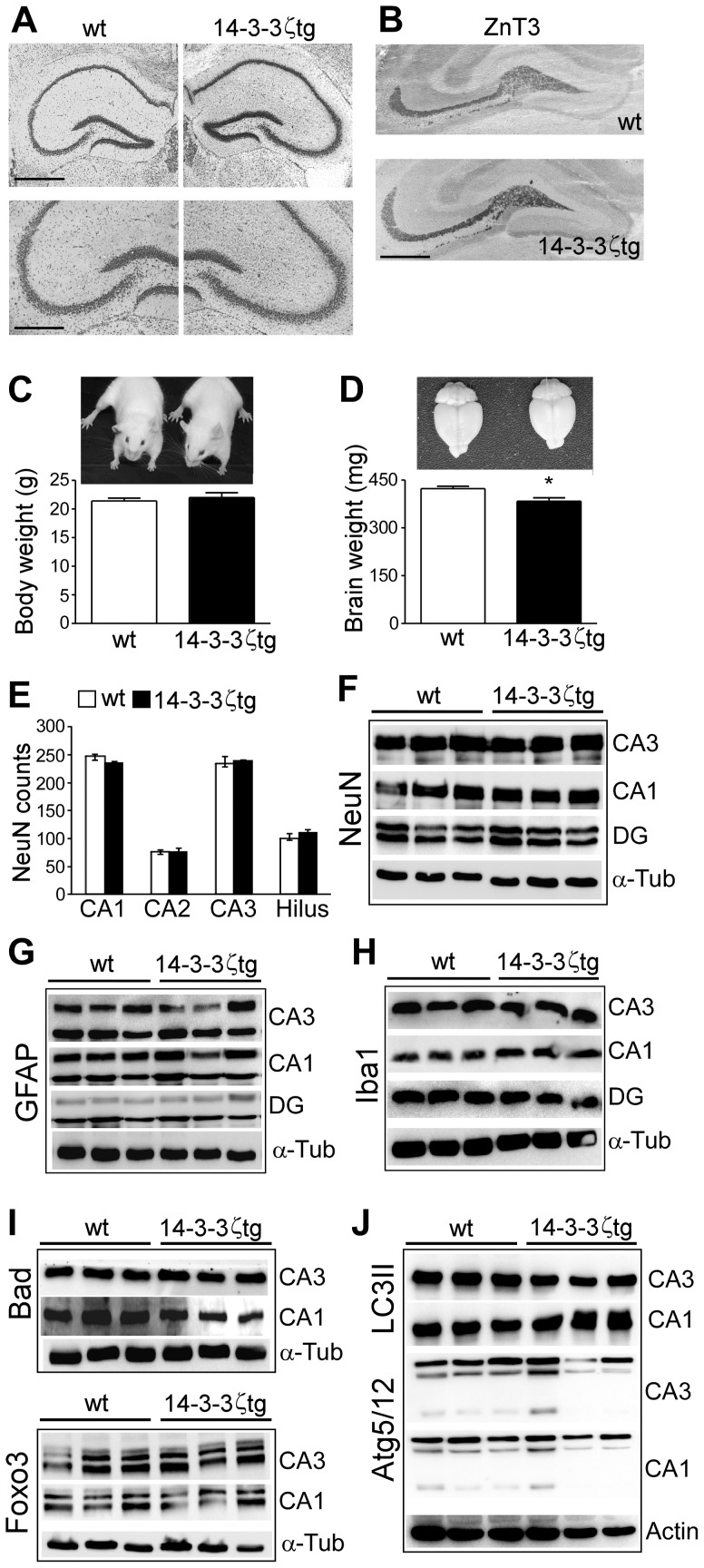
Hippocampal morphology and levels of apoptosis- and autophagy-related proteins in 14-3-3ζtg mice. (A) Nissl-stained sections of wt and 14-3-3ζtg mice. Scale bar, (top) 600 µm; (bottom), 330 µm. (B) Immunohistochemistry showing similar pattern of ZnT3 staining, a protein present in mossy fibers, in wt and 14-3-3ζtg mice. Scale bar, 500 µm. (C) Body weight in wt and 14-3-3ζtg mice at 6 weeks of age (*n* = 8 per group). (D) Brain weight in wt and 14-3-3ζtg mice at 6 weeks of age (*n* = 6 per group). *p<0.05 compared to wt. (E) NeuN counts in hippocampal subfields from sections of dorsal hippocampus from wt and 14-3-3ζtg mice (*n* = 6 per group). (F-J) Representative western blots (*n* = 1 per lane) showing similar levels of (F) NeuN, (G) the astrocyte marker GFAP, (H) microglia marker Iba1, (I) apoptosis-associated 14-3-3 binding proteins and (J) autophagy-related 14-3-3 binding proteins, in adult wt and 14-3-3ζtg mice.

We next examined hippocampal morphology in 14-3-3ζtg mice. Nissl-stained sections of 14-3-3ζtg mice were indistinguishable from wild-type mice (Figure 2A). Staining for the zinc transporter protein ZnT3 confirmed 14-3-3ζtg mice have a normal distribution of mossy fibers (Figure 2B). Mouse body weight was normal although brain weight was slightly lower (Figure 2C, D). Immunostaining and western blotting for the mature neuron marker NeuN determined hippocampal neuron distribution and counts were normal in the 14-3-3ζtg mice (Figure 2E, F). No differences were observed for levels of the astrocyte marker GFAP or the microglia marker Iba1 (Figure 2G, H).

### Normal Expression of Apoptosis-associated Proteins in 14-3-3ζtg Mouse Hippocampus

14-3-3 proteins oppose apoptosis by sequestering pro-apoptotic proteins including Bad, the transcription factor Foxo3 and ASK-1 [2], and recent work suggests 14-3-3 may also regulate autophagy [37], [38]. Western blot analysis of microdissected hippocampal subfields from 14-3-3ζtg mice found no differences from wild-type for levels of Bad, Foxo3, Bax or Bim (Figure 2I and data not shown), or any differences in autophagy-related proteins LC3II or Atg5/12 [38] (Figure 2J).

### Reduction in ER Chaperones and UPR Proteins in 14-3-3ζtg Mice

**Figure 3:**
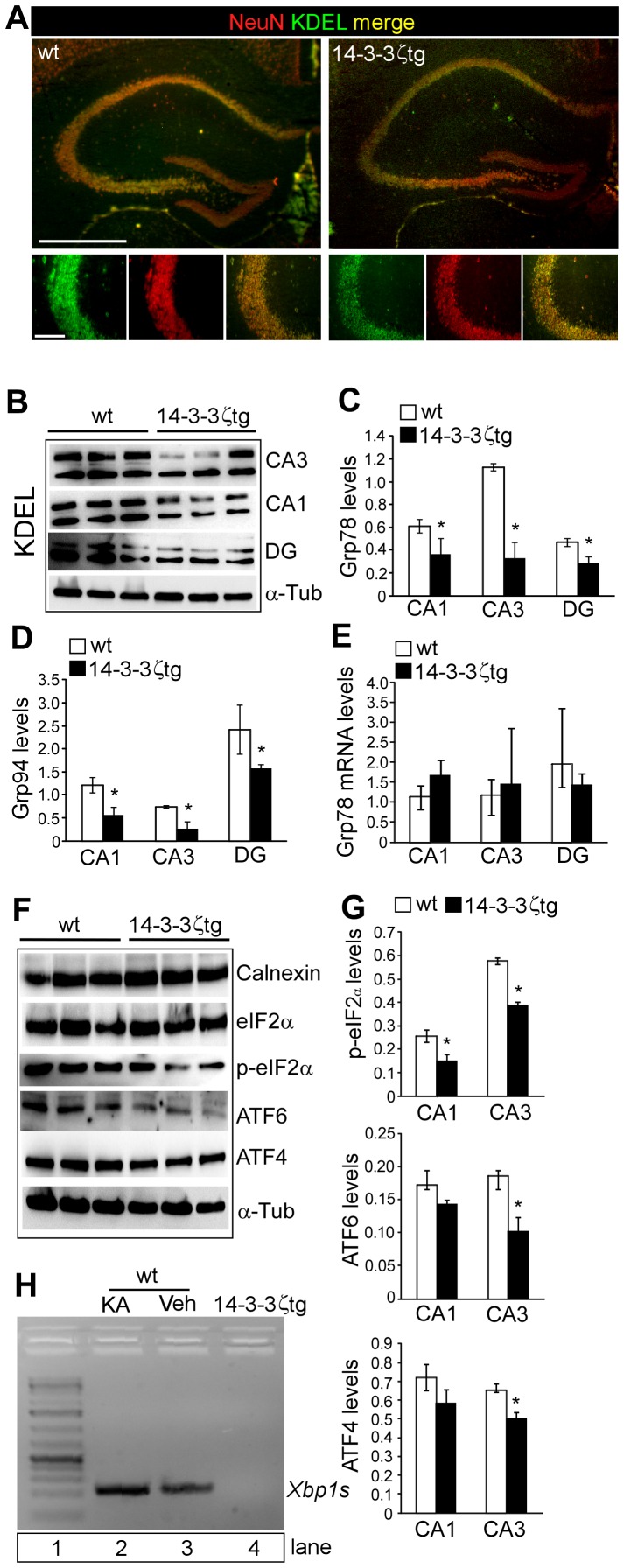
Reduced levels of ER and UPR-related proteins in 14-3-3ζtg mice. (A) Representative KDEL (green) immunostaining for wt and 14-3-3ζtg mice hippocampus. Neurons are identified by NeuN counterstaining (red). Merged panels confirm staining is neuronal. Note, lower KDEL immunoreactivity in 14-3-3ζtg mice. Scale bar (top), 700 µm; (bottom), 150 µm. (B) Representative western blots (*n* = 1 per lane) showing KDEL-containing proteins in microdissected subfields of wt and 14-3-3ζtg mice. (C, D) Basal Grp78 and Grp94 protein levels in wt and 14-3-3ζtg mice (*n* = 3 per group). (E) Real-time PCR measurement of *Grp78* levels in wt and 14-3-3ζtg mice in each subfield (*n* = 3 per group). (F) Western blots (*n* = 1 per lane) showing levels of various ER-related proteins in the CA3 subfield of 14-3-3ζtg mice compared to wt mice. (G) Graphs showing lower basal levels of p-eIF2α, ATF6 and ATF4 in select hippocampal subfields (*n* = 3 per group). (H) Gel showing levels of the spiced form of *Xbp1* from hippocampal lysates of; (lane 2) WT mice subject to seizures levels, (lane 3) Wt mice, (lane 4) 14-3-3ζtg mice. Lane 1 is a ladder. *p<0.05 compared to wt.

RNAi-mediated down-regulation of 14-3-3ζ in organotypic hippocampal cultures results in an ER stress-like response that features increased levels of ER chaperones, including Lys-Asp-Glu-Leu (KDEL)-containing proteins such as Grp78 and Grp94 [17]. To investigate whether overexpression of 14-3-3ζ alters ER chaperones or the UPR *in vivo* we began by staining tissue sections from wild-type and 14-3-3ζtg mice with antibodies against KDEL (Figure 3A). KDEL immunoreactivity was detected in all CA neuronal populations and in the granule neurons of the dentate gyrus in wild-type mice. Lower KDEL immunoreactivity was apparent in hippocampal sections from 14-3-3ζtg (Figure 3A). This difference was confirmed using western blot analysis of lysates from CA1, CA3 and the dentate gyrus-enriched portions of the hippocampus (Figure 3B-D). Basal mRNA levels of Grp78 and Grp94 were not different between wild-type and 14-3-3ζtg mice (Figure 3E and data not shown).

No differences were found between wild-type and 14-3-3ζtg mice for CA3, CA1 or dentate gyrus levels of calnexin or eIF2α (Figure 3F). In contrast, levels of phospho-eIF2α, ATF4 and ATF6 were lower in the CA3 subfield of 14-3-3ζtg mice (Figure 3F, G), although levels of phospho-eIF2α and ATF4 and ATF6 were not consistently different from wild-type in the dentate gyrus or CA1 subfield (Figure 3F, G and data not shown). Levels of the spliced transcript of *Xbp1* were not detectable in 14-3-3ζtg mice whereas low levels of this were detected in wild-type animals, which were increased in mice subject to seizures (Figure 3H).

### 14-3-3ζ Overexpression Protects Against ER Stress-induced Neuronal Death *in vivo*

**Figure 4:**
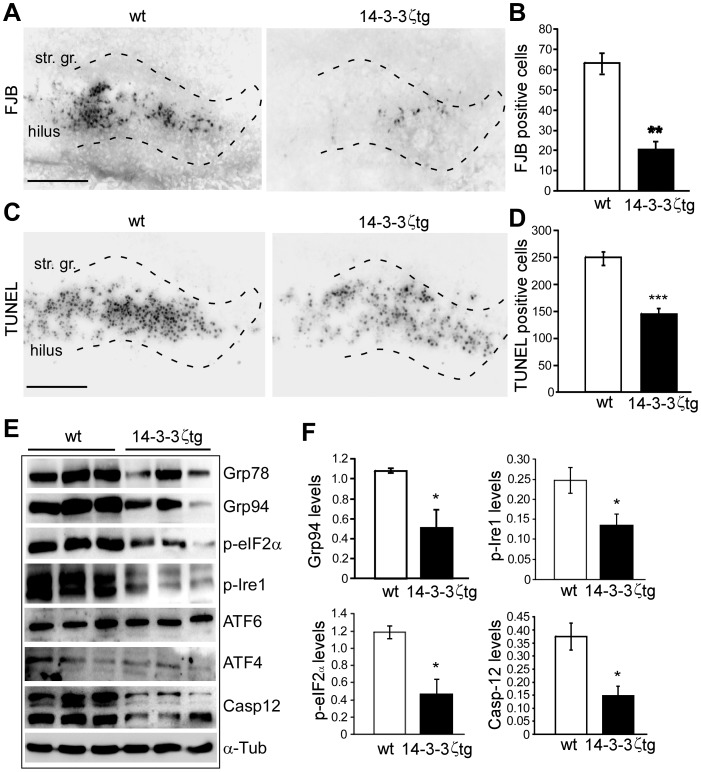
14-3-3ζtg mice are protected against ER stress-induced neuronal death *in vivo*. (A) Representative FJB staining of wt and 14-3-3ζtg mouse hippocampus 48 h after i.c.v. injection of tunicamycin (1 µl, 50 µM). Scale bar, 150 µm. Dotted lines depict upper and lower blades of the granule cell layer. str. gr., stratum granulosum. (B) Counts of FJB-positive cells 48 h after tunicamycin in wt and 14-3-3ζtg mice (*n* = 5 per group; **p<0.01 compared to wt). (C, D) Representative photomicrographs showing TUNEL staining in wt and 14-3-3ζtg mice 48 h after tunicamycin injection and graph quantifying the difference (*n* = 5 per group; ***p<0.001 compared to wt. (E, F) Representative western blots (*n* = 1 per lane) and semi-quantification of UPR and ER-associated protein levels between wt and 14-3-3ζtg mice (*n* = 3 per group; *p<0.05 compared to wt).

In view of the lower levels of ER stress/UPR-related proteins, we postulated that 14-3-3ζtg mice may show altered vulnerability to ER stress *in vivo*. To test this idea, wild-type and 14-3-3ζtg mice were given an intracerebroventricular injection of the ER stressor tunicamycin. In wild-type mice this resulted in extensive death of dentate granule neurons 48 h later, as detected by Fluor-Jade B (FJB) staining (Figure 4A) and TUNEL staining (Figure 4C). In contrast, tunicamycin-induced neuronal death was significantly reduced in 14-3-3ζtg mice (Figure 4B, D). This was despite lower levels of many ER/UPR-related proteins including Grp94, phospho-eIF2α and phospho-Ire1 and lower levels of caspase-12 (Figure 4E, F). ATF4 and ATF6 levels were not different after tunicamycin (Figure 4E).

### Seizure Severity is not Altered by 14-3-3ζ Overexpression in Mice

**Figure 5:**
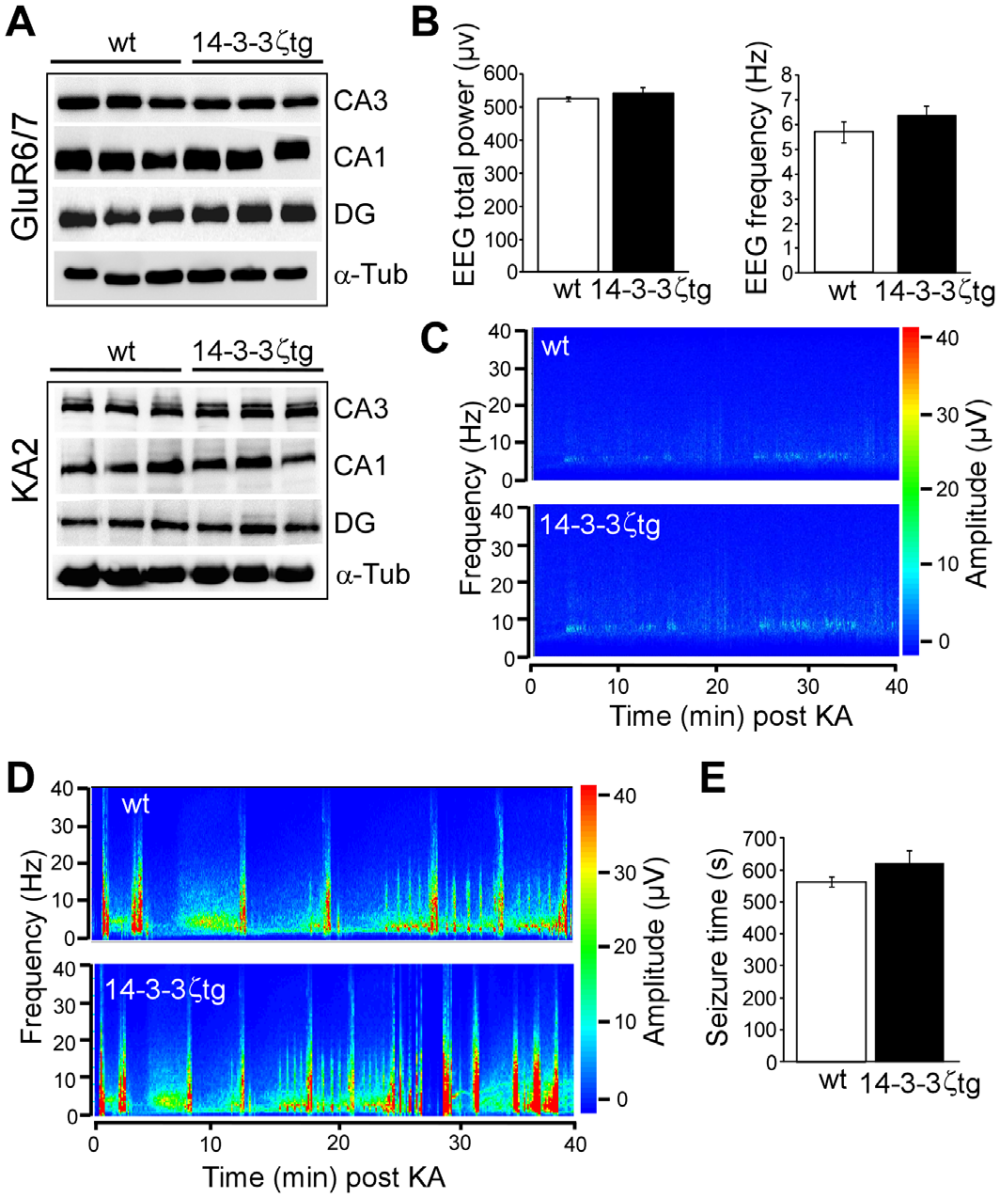
Baseline and seizure EEG in 14-3-3ζtg mice. (A). Protein levels of the kainic acid receptors GluR6/7 and KA2 in microdissected subfields of hippocampus from wt and 14-3-3ζtg mice. (B) Analysis of baseline EEG parameters during 40 min recordings from skull of wt and 14-3-3ζtg mice. No differences were detected between genotypes (*n* = 6 per group). (C) Representative EEG spectral activity plot of baseline EEG in wt and 14-3-3ζtg mice. (D, E) Representative spectral activity plot of EEG frequency and amplitude, and quantitative analysis of seizure duration (high amplitude and high frequency discharges) for wt and 14-3-3ζtg mice during the 40 min after intra-amygdala microinjection of kainic acid. No differences were detected between genotypes (*n* = 6-7 per group).

Reduced 14-3-3ζ levels have been shown to increase vulnerability to kainic acid in organotypic hippocampal slice cultures [17]. To determine whether over-expression of 14-3-3ζ has effects on seizure-induced neuronal death *in vivo* we examined the response of mice to status epilepticus. Intra-amygdala microinjection of kainic acid produces prolonged seizures which spread to the hippocampus via the entorhinal cortex and perforant pathway. Mossy fibres from dentate granule neurons synapse directly on CA3 pyramidal neurons, which are particular vulnerable to seizure-induced neuronal death in this model [31]. Protein levels of kainic acid receptors appeared normal in 14-3-3ζtg (Figure 5A). Forty minute EEG recordings from skull-mounted electrodes detected no differences in baseline total power or frequency parameters in 14-3-3ζtg mice (Figure 5B, C). Next, we recorded seizures in wild-type and 14-3-3ζtg mice. Seizure durations in wild-type mice were similar to those reported previously for SJL mice in this model [31]. Electrographic seizure EEG was not different in 14-3-3ζtg mice (Figure 5D, E).

### 14-3-3ζ Overexpression Protects Against Seizure-induced Neuronal Death *in vivo*

**Figure 6:**
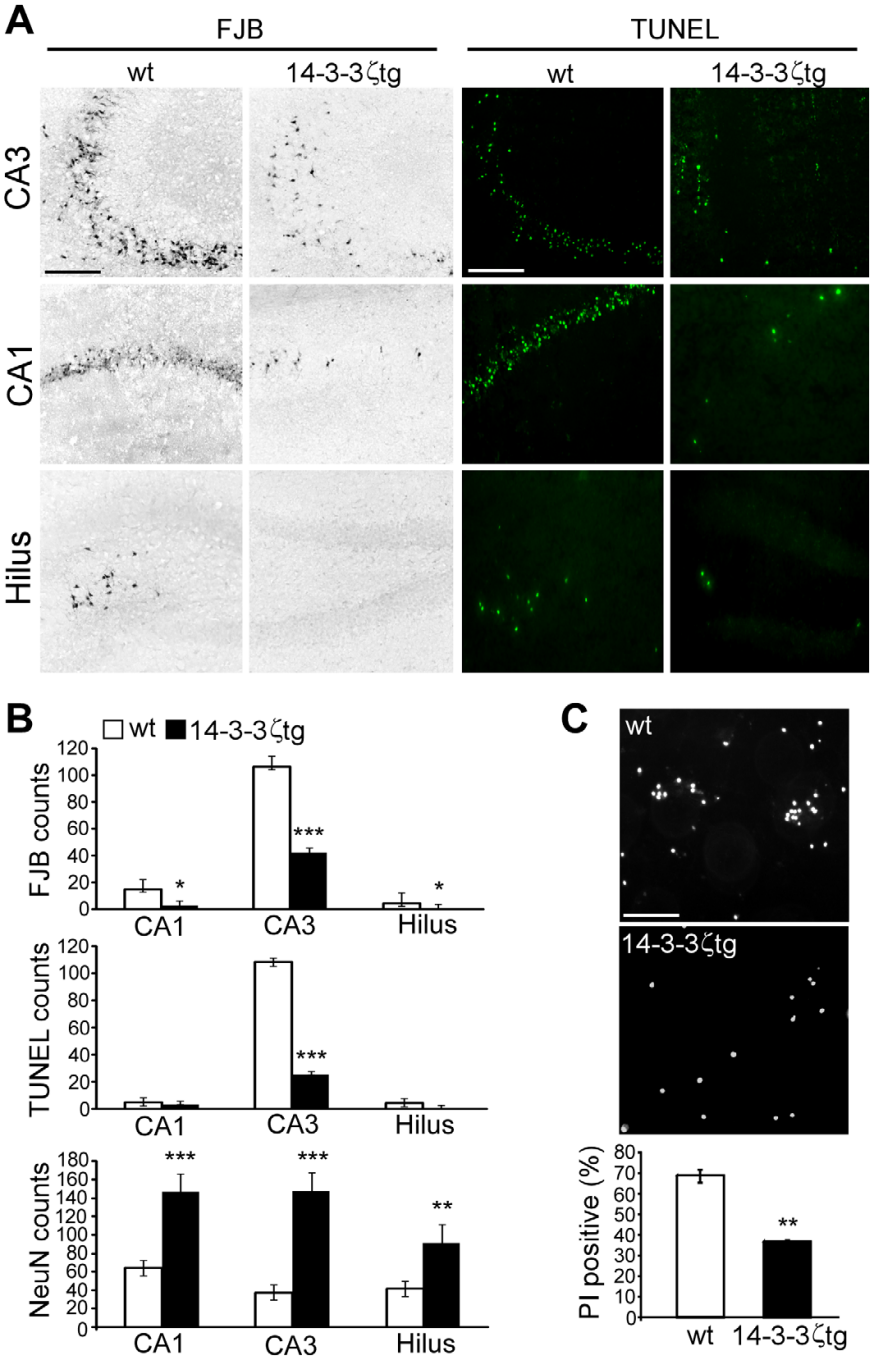
14-3-3ζtg mice are protected against seizure-induced neuronal death *in vivo* and *in vitro*. (A) Representative FJB and TUNEL staining for wt and 14-3-3ζtg mice 72 h after status epilepticus for the CA1, CA3 and hilar regions. Scale bar, 120 µm. (B) Semi-quantification of seizure damage and neuron survival (NeuN counts) for wt and 14-3-3ζtg mice (*n* = 6-10 per group). (C) Primary cultures of hippocampal neurons from wt and 14-3-3ζtg mice were treated with kainic acid and then cell death determined as percentage propidium iodide (PI) positive. (Panels above) Representative photomicrographs of PI-stained neurons 24 h after KA treatment. Scale bar, 25 µm. Graph shows reduced cell death in 14-3-3ζtg mice (*n* = 3 per group). *p<0.05; **p<0.01; ***p<0.001 compared to wt.

Hippocampal damage in SJL mice subjected to intra-amygdala kainic acid-induced status epilepticus is found extensively in the CA3 subfield, with additional neuronal death in the CA1 subfield and hilus [31]. We next examined seizure-damage 72 h after status epilepticus in wild-type and 14-3-3ζtg mice. As expected, FJB staining of tissue sections from wild-type mice revealed hippocampal damage was most extensive in the ipsilateral CA3 subfield (Figure 6A). Seizure damage was also present in the CA1 subfield and hilus (Figure 6A). FJB-staining was reduced in 14-3-3ζtg mice in all subfields (Figure 6A, B). Supporting these results, staining for irreversible DNA fragmentation using TUNEL confirmed extensive neuronal death in the CA3, CA1 and hilus of wild-type mice, which was reduced in 14-3-3ζtg mice (Figure 6A, B). 14-3-3ζtg mice also displayed higher levels of normal NeuN staining in each hippocampal subfield (Figure 6B and data not shown). Western blot analysis of protein lysates from wt and 14-3-3ζtg mice revealed significantly lower levels of several proteins related to ER stress and the UPR in the CA3 subfield, including Grp 78 and 94, phospho-eIF2α and caspase-12 (*p*<0.05; *n* = 3 per group, data not shown).

Finally, to support the *in vivo* seizure data we exposed primary cultures of hippocampal neurons to kainic acid, an *in vitro* model of excitotoxicity [34]. Kainic acid treatment produced ∼70% cell death in primary hippocampal cultures from wild-type SJL mice. In contrast, cell death was significantly lower in primary hippocampal cultures from 14-3-3ζtg mice (Figure 6C).

## Discussion

In the present study we report that transgenic overexpression of 14-3-3ζ in mice alters basal levels of proteins involved in the UPR pathway. 14-3-3ζ overexpression nevertheless potently protected the hippocampus against ER stress and status epilepticus *in vivo*. These results suggest that restitution or delivery of 14-3-3ζ may protect against neurologic or neurodegenerative injuries in which excitotoxicity, ER stress or an impaired UPR response is a causal patho-mechanism.

14-3-3 proteins are increasingly recognized as crucial molecular chaperones during brain development, in neuronal function and disease [2]. There is some functional redundancy among certain 14-3-3 isoforms [39], although loss of 14-3-3ζ does not appear to be compensated by increased levels of other isoforms [5], [6]. Likewise, 14-3-3 isoforms display selectivity in their capacity to prevent neuronal death according to the nature of the stressor, which may derive from their interacting partners and subcellular distribution [40]. The present study is the first to characterize the brain phenotype of mice overexpressing 14-3-3ζ. The animals were originally developed to explore tumor-promoting effects of 14-3-3ζ overexpression [30] and the mice do develop tumors but beyond the time when mice were used presently (E. J-M., unpublished data). The overexpressed 14-3-3ζ was found throughout the mouse hippocampus and detectable in the cytosol, nucleus, mitochondria and ER-containing fractions. Thus, the transgene expressed and distributed similarly to the endogenous protein. This excludes features of the phenotype of the mice being due to erroneous distribution of extra 14-3-3ζ to cell populations or compartments in which the protein is not ordinarily present. 14-3-3ζ protein was recently reported to be restricted to the CA pyramidal neurons and granule neurons of the hippocampus in adult mice [6], [15], [17]. Our studies confirmed these cells were immunoreactive for 14-3-3ζ, and expressed the myc-tagged transgene, but we detected the transgene in other brain regions, consistent with other studies showing 14-3-3ζ to be present outside the hippocampus in adult brain [41].

14-3-3ζ has been reported to regulate neurite outgrowth and is required for normal hippocampal development [6], [42]. Indeed, deletion of 14-3-3ζ results in mis-located pyramidal neurons and changes to the mossy fiber pathway within the infra- and supra-pyramidal layer and a predicted increased excitatory drive onto CA3 pyramidal neurons [6]. We did not detect any hippocampal abnormalities in 14-3-3ζtg mice, indicating that overexpression of 14-3-3ζ does not produce an opposite phenotype to gene deletion. We also detected no differences in body weight, reported for 14-3-3ζ^−/−^ mice [6] although a smaller brain weight was noted. The cause of this is uncertain and differences in neuron or glia populations were not observed. Indeed, consistent with normal brain development, we found no differences in baseline or seizure EEG in 14-3-3ζ transgenic mice, establishing an equivalent episode of status epilepticus was likely incurred and thus differences in damage can be confidently assigned to altered cell death/survival signalling rather than due to reduced seizures.

14-3-3 proteins regulate apoptosis by sequestering pro-apoptotic proteins including Bad and ASK-1, which are activated by excitotoxic insults to the brain and contribute to neuronal death in models of seizure and stroke [12], [43]. Overexpression of 14-3-3ζ did not alter resting levels of apoptosis-associated proteins to which it is known to bind, or change levels of autophagy-related proteins. Kainic acid receptors, which are trafficked from the ER by a 14-3-3-regulated mechanism [4], were also at normal levels in 14-3-3ζtg mice. Nevertheless, additional 14-3-3ζ may function as a pool to buffer against pro-apoptotic proteins released from endogenous 14-3-3ζ or other isoforms during apoptosis. The main molecular adjustment in 14-3-3ζtg mice, however, was downregulation of proteins involved in the UPR pathway. Most prominent was reduced levels of KDEL-containing proteins although all branches of the UPR were affected. This was probably due to post-translational mechanisms since transcript levels of several tested genes were normal. 14-3-3ζ overexpression did have effects on mRNA, however, reducing *Xbp1* splicing, a consequence of the activation of the IRE1 branch of the UPR that promotes increased expression of ER chaperones [22]. Taken together, these data suggest overexpressed 14-3-3ζ may produce a selective adjustment of the UPR. Since this included reduced levels of proteins involved in folding, this is consistent with 14-3-3ζ functioning as a sweeper of mis-folded proteins [3].

ER stress is implicated as a patho-mechanism underlying neurodegeneration in several diseases, including epilepsy [44], although NMDA receptor-induced neuronal death can occur independently of ER stress *in vivo* [45]. Studies here demonstrated that overexpressed 14-3-3ζwas capable of protecting against ER stress induced by tunicamycin. Tunicamycin causes ER stress by preventing N-glycosylation of proteins, thus resulting in a build-up of proteins in the ER and triggering the UPR and ER stress-induced apoptosis, although direct effects on neurotransmission have been reported [46]. Tunicamycin injection into the brain caused the selective death of neurons within the dentate gyrus, consistent with *in vitro* reports [17]. 14-3-3ζ strongly protected against tunicamycin-induced neuronal death despite the lower resting levels of UPR/ER stress proteins. Even after treatment, levels of many UPR/ER stress proteins remained lower in 14-3-3ζtg mice, excluding an effect secondary to normalization of levels. Higher 14-3-3ζ levels may therefore reduce the stress caused by tunicamycin and over-expressed 14-3-3ζ may be sufficient to protect in the absence of a complete complement of ER stress chaperones. 14-3-3ζ delivery may be a means to reduce ER stress where the normal UPR response is inadequate or otherwise disrupted, such as in certain neurologic and neurodegenerative diseases [47]. The data also compliment the findings of 14-3-3ζ silencing in hippocampus, which triggers up-regulation of KDEL-containing proteins and sensitizes against tunicamycin-induced cell death [48].

A major finding in the present study was that overexpression of 14-3-3ζ potently protected against seizure-induced neuronal death *in vivo*. Protection was found for both pyramidal and hilar interneurons, indicating 14-3-3ζ overexpression protects regardless of neuronal phenotype or location within the hippocampal circuitry. These results compliment earlier work that showed lowering 14-3-3ζ levels in the mouse hippocampus increased neuronal death after kainate treatment of organotypic cultures [48]. The extent of protection is similar and in several cases greater than achieved by deletion of pro-apoptotic members of the Bcl-2 family in the same model [49], [50], [51]. This would be consistent with 14-3-3ζ function either upstream or being involved in curtailing cell death via actions in more than one compartment. Over-producing an anti-apoptotic protein may also be more effective than deleting a pro-apoptotic protein. Again, the protection obtained was in spite of a lower compliment of ER stress chaperones in these mice. This agrees with *in vivo* evidence that NMDA-dependent neuronal death *in vivo* is not ER stress-dependent [45]. This could mean that either 14-3-3ζ is more effective than these proteins at protection in this model, or that the protection derives from functions in addition to ER stress-related cell death. This is likely; we detected 14-3-3ζ overexpression throughout the cell and 14-3-3 is able to sequester various pro-apoptotic Bcl-2 family proteins which contribute to seizure-induced neuronal death in the model [12], [52]. Whether or not the reduced hippocampal damage in 14-3-3ζtγ mice would result in a beneficial effect on the post-status epilepticus epilepsy phenotype is uncertain, although our previous studies in which hippocampal damage was reduced by genetic or other means, led to fewer spontaneous recurrent seizures [51], [53].

Levels of several proteins associated with the UPR have been found to be higher in the hippocampus of patients with temporal lobe epilepsy [26], [27]. Levels of these proteins were generally lower, however, in the hippocampus of 14-3-3ζ transgenic mice suggesting the model does not phenocopy this molecular feature of human temporal lobe epilepsy. Nevertheless, elevated 14-3-3ζ levels were reported in the microsome-containing fraction of hippocampus from patients with temporal lobe epilepsy [15]. Although we cannot directly compare the higher 14-3-3ζ levels in the transgenic mice with those in patient brain, we may speculate, on the basis of our results here, that increased 14-3-3ζ in intractable human epilepsy could be protective against ongoing neuron loss. We note, however, that 14-3-3ζ is not uniformly neuroprotective, and fails to prevent neurodegeneration in models of Parkinson’s disease [40]. 14-3-3ζ can also promote phosphorylation of Tau and chronic over-expression might have potentially deleterious effects on the brain [54], [55], which could be assessed using the present model. Any strategy to enhance 14-3-3ζ expression may provide neuroprotection only against the acute effects of prolonged seizures or perhaps stroke, which share common patho-mechanisms such as excitotoxicity and apoptosis-associated signalling [11].

In summary, the present study demonstrates that 14-3-3ζ overexpression results in a selective downregulation of UPR pathways and confers protection against ER stress- and seizure-induced neuronal death in the mouse hippocampus. Restitution or overexpression of this 14-3-3 isoform may be a potential therapeutic approach for status epilepticus but not necessarily all CNS diseases associated with impaired 14-3-3ζ expression [2].
